# Multipotent mesenchymal stem cells in lung fibrosis

**DOI:** 10.1371/journal.pone.0181946

**Published:** 2017-08-21

**Authors:** Katrin E. Hostettler, Amiq Gazdhar, Petra Khan, Spasenija Savic, Luca Tamo, Didier Lardinois, Michael Roth, Michael Tamm, Thomas Geiser

**Affiliations:** 1 Department of Biomedicine, University Hospital Basel, University of Basel, Basel, Switzerland; 2 Clinics of Respiratory Medicine, University Hospital Basel, University of Basel, Basel, Switzerland; 3 Division of Pulmonary Medicine, University Hospital Bern, Bern, Switzerland; 4 Department of Pathology, University Hospital Basel, University of Basel, Basel, Switzerland; 5 Division of Thoracic Surgery, University Hospital Basel, University of Basel, Basel, Switzerland; Children's Hospital of Los Angeles, UNITED STATES

## Abstract

**Rationale:**

Stem cells have been identified in the human lung; however, their role in lung disease is not clear. We aimed to isolate mesenchymal stem cells (MSC) from human lung tissue and to study their *in vitro* properties.

**Methods:**

MSC were cultured from lung tissue obtained from patients with fibrotic lung diseases (n = 17), from emphysema (n = 12), and normal lungs (n = 3). Immunofluorescence stainings were used to characterize MSC. The effect of MSC-conditioned media (MSC-CM) on fibroblast proliferation and on lung epithelial wound repair was studied.

**Results:**

Expression of CD44, CD90, and CD105 characterized the cells as MSC. Moreover, the cells stained positive for the pluripotency markers Oct3/4 and Nanog. Positive co-stainings of chemokine receptor type 4 (CXCR4) with CD44, CD90 or CD105 indicated the cells are of bone marrow origin. MSC-CM significantly inhibited the proliferation of lung fibroblasts by 29% (p = 0.0001). Lung epithelial repair was markedly increased in the presence of MSC-CM (+ 32%). Significantly more MSC were obtained from fibrotic lungs than from emphysema or control lungs.

**Conclusions:**

Our study demonstrates enhanced numbers of MSC in fibrotic lung tissue as compared to emphysema and normal lung. The cells inhibit the proliferation of fibroblasts and enhance epithelial repair *in vitro*. Further *in vivo* studies are needed to elucidate their potential role in the treatment of lung fibrosis.

## Introduction

Interstitial lung diseases (ILD) are a heterogeneous group of disorders and pulmonary fibrosis is the common end stage of many ILDs. They are characterized by an excessive deposition of extracellular matrix (ECM) by fibroblasts leading to the destruction of the lung architecture [1, 2]. The most common and aggressive form of ILD is idiopathic pulmonary fibrosis (IPF) having a 5-year survival of only 20% [2]. The pathomechanisms of IPF are incompletely understood but repetitive micro-injuries to alveolar epithelial cells and dysregulated alveolar wound repair with impaired re-epithelialization are regarded as the initial processes [3, 4]. Data showing the induction of pulmonary fibrosis by targeted injury of alveolar epithelial cells support this concept [3]. While previous therapeutic approaches mainly targeted fibroblast proliferation and ECM deposition, newer strategies are aimed at replacing damaged epithelial cells and restoring normal repair processes [2]. Accordingly, the use of stem cells to improve regeneration and reduce fibrosis has been reported in animal models of lung fibrosis [5–9]. Very recently, three phase 1 clinical trials demonstrated the feasibility and safety of administered mesenchymal stromal/stem cells in IPF patients [10–12]. We have demonstrated previously the presence of bone marrow derived, hepatocyte growth factor (HGF) -secreting, stem cells in human lung tissue from patients with usual interstitial pneumonia [13]. Furthermore, HGF-expressing bone marrow derived stromal cells (BMSC) attenuated bleomycin induced pulmonary fibrosis in the rat lung [13], suggesting that these cells have anti-fibrotic properties.

Endogenous stem/progenitor cells have been reported in the human lungs and are thought to help in repair and regeneration [14]. However, no comparative study has been performed to isolate and characterize these endogenous stem/progenitor cells from different lung diseases. We therefore aimed at isolating endogenous stem/progenitor cells from healthy, emphysematous and fibrotic lungs and to ascertain their phenotype, origin and possible biological role specifically in fibrotic lungs.

In the present study, we demonstrate the presence of potential mesenchymal stem cells (MSC) in fibrotic human lung, and provide evidence for their anti-fibrotic properties *in vitro*.

## Materials and methods

### Ethical approval

The Human Ethics Committee of the University of Basel approved the study (EKBB 05/06). Human lung tissue was obtained with approval of the Human Ethics Committee of the University of Basel (EKBB 05/06) and written informed consent was obtained from all patients who underwent lung biopsy.

### Patients

Between November 2011 and April 2014 cell cultures of primary lung cells were established from lung tissue obtained from 32 patients undergoing video-assisted thoracoscopic surgery (VATS) performed at the Division of Thoracic Surgery, or undergoing flexible bronchoscopy with transbronchial biopsy at the Clinics of Respiratory Medicine, University Hospital Basel, Switzerland. Patient characteristics with clinical diagnosis are summarized in Table 1. In patients with lung tumors, lung tissue for cell culture was obtained from the macroscopically normal part away from the tumor. A complete set of pulmonary function tests (PFT) within 2 weeks prior to lung biopsy was available from all patients. PFTs were performed by using body plethysmography and the carbon monoxide diffusion capacity (Jaeger, Wuerzburg, Germany). Tests were performed according to the European Respiratory Society standards [15, 16]. High resolution computed tomography (CT) scans of the lungs were performed in all patients prior to VATS/bronchoscopy.

**Table 1:** Baseline demographics, pulmonary function tests, indication for lung biopsy and final diagnosis of all 32 patients.

Patient number	Age	Sex	TLC absolute [l]	TLC percentage predicted	FVC absolute [l]	FVC percentage predicted	FEV1 absolute [l]	FEV1 percentage predicted	FEV1/VC max	DLCO absolute [mmol/min/kPa]	DLCO percentage predicted	Indication for lung biopsy	Final multidisciplinary diagnosis
1	80	m	7.1	107.9	1.9	58.1	0.8	32.9	29.6	2.4	32.3	Lung volume resection surgery	COPD with emphysema
2	47	f	5.3	108.3	3	95.3	2.4	92.5	80.8	4.7	56.7	Pulmonary nodule of unknown dignity	Hamartoma
3	65	f	4.1	78.5	1.6	57	1.6	66.9	82.2	2.4	31.8	Non-classified interstitial lung disease	Cryptogenic organizing pneumonia
4	34	f	3.5	78.3	2.4	76.5	2.3	83.1	93.9	7.6	90	Non-classified interstitial lung disease	Systemic sclerosis with lung involvement
5	57	m	9.1	117.6	3.5	71.7	1.7	45.1	38.7	7.7	71.2	Bronchiolitis obliterans syndrome after HSCT	Pulmonal GvHD with bronchiolitis obliterans after HSCT
6	29	m	4.8	67.4	2.2	43.1	1.2	27.5	50.6	8	67.8	Bronchiolitis obliterans syndrome after HSCT	Pulmonal GvHD with bronchiolitis obliterans after HSCT
7	41	m	6.8	93.4	2.6	52.4	1.4	34.8	42.3	8.7	77.9	Bronchiolitis obliterans syndrome after HSCT	Pulmonal GvHD with bronchiolitis obliterans after HSCT
8	81	m	3.9	61.9	2.1	64.4	1.7	69.9	72.7	3.3	45.4	Non-classified interstitial lung disease	Rheumathoid arthritis with lung involvement
9	47	f	4.7	112.1	2.9	100.8	2.4	108.4	73.1	5.5	80.5	Non-classified interstitial lung disease	Rheumathoid arthritis with lung involvement
10	56	m	9.4	132.9	3.4	77.5	1.1	31.2	26.3	5	50.6	Lung volume resection surgery	COPD with emphysema
11	51	m	7.4	123.9	2.1	57.5	0.5	18.7	21.2	2.9	33.6	Lung volume resection surgery	COPD with emphysema
12	55	m	4.7	66	2.1	47.5	1.8	51.3	73.9	6.2	60.9	Bronchiolitis obliterans syndrome after HSCT	Pulmonal GvHD with lymphocytic bronchiolitis after HSCT
13	66	f	5.7	139.5	1.5	76.8	0.6	37	35.6	1	17.1	Lung volume resection surgery	COPD with emphysema
14	68	f	8.2	180.4	1.4	61.1	0.5	27.8	29.1	1.8	27.9	Lung volume resection surgery	COPD with emphysema
15	65	m	6.3	90.4	2.8	68.1	1.9	62.5	60.5	7	75.9	Bronchiolitis obliterans syndrome after HSCT	Pulmonal GvHD with lymphocytic bronchiolitis after HSCT
16	70	m	6.1	89.4	3.1	80.6	2.4	82	75.1	6.9	79.4	Pulmonary nodule of unknown dignity	Lung metastasis of urothel carcinoma
17	73	m	9.8	142.8	3.4	90.1	0.9	31.2	23.9	1.4	17.1	Lung volume resection surgery	COPD with emphysema
18	55	f	7.9	155.7	2.2	75.6	0.5	22.4	21.7	2.3	29	Lung volume resection surgery	COPD with emphysema
19	79	m	6.1	91.9	3.4	97.7	2.4	93.6	70.2	4.1	55.6	Local recurrence of known NSCLC	Emphysema/NSCLC
20	66	m	9.2	142.3	2.2	60.7	0.6	23.6	25.9	3.6	34	Bullectomy	COPD with emphysma
21	65	m	8.1	139.2	3.6	111.4	0.9	38.4	25.5	3.2	42.3	Lung volume resection surgery	COPD with emphysema
22	59	f	5.7	105.6	3.3	105.3	2.4	93.4	75.3	5.6	67.6	Non-classified interstitial lung disease	Lupus erythematodes with lung involvement
23	84	f	3.9	95.5	2.5	152.9	2.2	170.6	80.6	2.6	48.3	Non-classified interstitial lung disease	Cryptogenic organizing pneumonia
24	72	f	6.4	138.8	1.5	70.1	0.7	38.3	31.1	1.8	27.7	Pulmonary nodule of unknown dignity	COPD with emphysema/NSCLC
25	72	f	5.4	117.9	2.2	100.2	1.4	75.3	56.3	3	45.3	Pulmonary nodule of unknown dignity	COPD with emphysema/NSCLC
26	74	f	4.8	95	2.8	117	2.4	121	83.4	3.1	44.1	Non-classified interstitial lung disease	Lupus erythematodes with lung involvement
27	61	f	5.3	109.6	2.6	97.5	1.9	87.7	70.1	7.2	96.3	Chronic cough of unknown origin	Chronic cough of unknown origin
28	72	m	4.3	60.2	2.8	70.8	2.4	79	73.8	3.7	41.9	Non-classified interstitial lung disease	Idiopathic pulmonary fibrosis
29	60	f	2.8	54.9	1.5	52.9	1	40.8	65.3	na	na	Non-classified interstitial lung disease	Idiopathic pulmonary fibrosis
30	59	m	6	76	3.5	72.3	2.8	72.7	70.5	4.1	37.6	Non-classified interstitial lung disease	Idiopathic pulmonary fibrosis
31	56	m	6.1	84	4.4	96	3.6	99	79.5	7.7	75.7	Non-classified interstitial lung disease	Idiopathic pulmonary fibrosis
32	60	m	4.7	72	3.4	87	2.9	93	83.8	3.2	35.1	Bronchiolitis obliterans syndrome after HSCT	Pulmonal GvHD with lymphocytic bronchiolitis after HSCT

Numbering of patients according to date of lung biopsy. COPD: chronic obstructive pulmonary disease; DLCO: diffusion capacity of the lung for carbon monoxide; FEV1: forced expiratory volume 1 second; FVC: forced vital capacity; GvHD: graft-versus-host disease; HSCT: hematopoietic stem cell transplantation; NSCLC: non-small cell lung cancer; TLC: total lung volume.

### Cell culture

Lung tissue was cut into small pieces and placed into cell culture flasks. MSC were grown under standard conditions (37°C, 21% O_2_, 5% CO_2_) in RPMI medium supplemented with 10% fetal calf serum (FCS), 20 U/L penicillin, 20 μg/ml streptomycin and 2.5 μg/ml amphotericin B was used. Cultures were controlled five times a week for outgrowth of cells by sprouting. MSC-growth was considered as “positive” when cells formed a confluent cell layer around the biopsy. If there was no growth of MSC within fourteen days after start of cell culture the biopsy was rated as “negative”. To obtain conditioned medium (CM) from cells, fresh cell culture medium was added to non-passaged MSC and incubated for 24 hours (37°C, 5% CO_2_). The CM was collected, centrifuged (2000 x g, 5 minutes) to remove any cell debris, split into aliquots and stored at– 20°C. CM derived from the same cell line was pooled. Fibroblasts were cultured as described earlier [17]. For the culture of primary human alveolar epithelial type II cells pieces of lung tissue were placed into cell culture flasks for cell sprouting containing supplemented epithelial growth medium (Cnt-17) (CELLnTEC Advanced Cell System AB; Bern, Switzerland). Complete epithelial culture medium was replaced every fourth day.

### Paraffin embedded lung slices

Lung tissue sections from selected patients diagnosed with IPF/usual interstitial pneumonia (UIP) or chronic hypersensitivity pneumonitis were studied. Sections with normal lung served as controls.

### Immunofluorescence stainings and immunohistochemistry in cultured cells and lung tissue sections

Immunofluorescence analysis of confluent, non-passaged cells was performed as previously described [17]: confluent, non-passaged cells were fixed with 4% formalin (10 minutes), and permeabilized with methanol/acetic acid (3:1, ice-cold, 10 minutes). Cells were then blocked with 5% BSA (1 hour), and incubated with antibody against fibronectin (SantaCruz, LabForce AG; Nunningen, Switzerland), α-smooth muscle actin (α-SMA) (Epitomics, LabForce AG), or E-cadherin (Santa Cruz, LabForce AG) for 1 hour. The primary antibodies were detected by addition of fluorescein-conjugated donkey anti-goat (Southern Biotech, BioConcept; Allschwil, Switzerland) or cy3-conjugated goat anti-rabbit antibody (Invitrogen; Lucerne, Switzerland) for 1 hr. To visualise the nuclei 4,6-diamido-2-phenylindole (Sigma) was added (5 min) and cells were subsequently examined on a fluorescence microscope. For Nanog (R&D systems) and Oct3/4 (Abcam, USA) stainings cells were fixed as described above, and incubated with primary antibodies over night at 4°C. Next day the cells were treated with appropriate secondary antibodies and visualized using Leica DMI 4000 B. The different fluorescent labelled antibodies used were FITC (for Oct3/4) and Alexa Fluor 594 (for Nanog) (ThermoFisher Scientific, Life Technologies Europe; Zug, Switzerland). Immunofluorescence stainings for CD90, CD105, CXCR4 and immunohistochemistry for CD44 and CXCR4 were performed as described previously [13].

### Real-time RT-PCR

Total RNA was extracted with a Quick-RNA MiniPrep Kit (ZymoResearch, Orange, CA). RNA levels were determined by real-time PCR using an E-Cadherin (Hs01023894), Oct3/4 (Hs01895061_u1), NANOG (Hs02387400_g1) or GAPDH (Hs03929097_g1) TaqMan® Gene Expression Assay (both Applied Biosystems, Foster City, CA). Samples were run at 50°C for 2 min, 1 cycle; 95°C for 10 min, 1 cycle; 95°C for 15 s, 60°C for 1 min, 40 cycle and quantified by the ΔΔCt calculation method.

### Assay for mesenchymal differentiation

Cells were stimulated with specific differentiation media Stem Pro differentiation kits (Life technologies, USA) following manufactures protocol. For myogenic differentiation two different media were prepared, cells were grown in DMEM media with 2% horse serum (Life technologies, USA) and 1% l-glutamin (Life technologies, USA) for 3 days and then changed to DMEM with 2% horse serum, 1%l-glutamin 1ng/ml bFGF (Peprotech, USA) and 0.4μg/ml dexamethasone (Sigma Aldrich, USA). Adipogenic differentiation was assessed with Red oil O (Sigma Aldrich, USA) staining for fat vacuoles; Myogenic differentiation was assessed using staining for α-SMA; Osteogenic differentiation was demonstrated by assessing alkaline phosphotase activity (BCIP/NBT, Thermo Scientific, USA); Chondrogenic differentiation was demonstrated via staining for Toluidine Blue (Sigma Aldrich, USA).

### Fibroblast proliferation

Cells were seeded (10^4^ cells/ml) in 24-well plates, grown until 80% confluence, and serum-deprived for 24 hours (0.1% FCS). Fibroblasts were incubated with MSC-CM for 48 hours before being automatically counted (Coulter, particle counter).

### Hepatocyte growth factor analysis

HGF-levels in MSC-CM were quantified by enzyme-linked immunoabsorbent assay (ELISA) kit as instructed (R&D systems, UK).

### *In vitro* alveolar epithelial wound repair assay

Wound repair assay was performed as reported previously [18]: human alveolar epithelial-like cells A549 (American Type Culture Collection [ACCT]; Rockville, MD, United States of America) were cultured to confluence in six-well plates in RPMI supplemented with 10% FCS. The cell layer was mechanically wounded using a pipette tip, and CM obtained from mesenchymal stem cells with and without different concentrations of HGF-neutralizing antibodies (0.1, 0.4, 0.8 ng/ml) was added to the wounded cells. Images of the wound surface were captured at time 0 and after 24 hours using a microscope (Leitz Diavert, Wetzlar, Germany) connected to a digital camera (Nikon Coolpix). Image J software (NIH, USA) was applied to analyze the wound surface and wound repair was expressed as percentage of lung epithelial wound closure after 24 hours.

### Statistical analysis

Statistical comparisons were made by using Student’s t-test. p-values ≤ 0.05 were considered significant. Where applicable, data are shown as mean ± standard error of the mean (SEM) from at least three independent experiments.

## Results and discussion

### Culture and characterization of undifferentiated cells from adult human lungs

**Fig 1:**
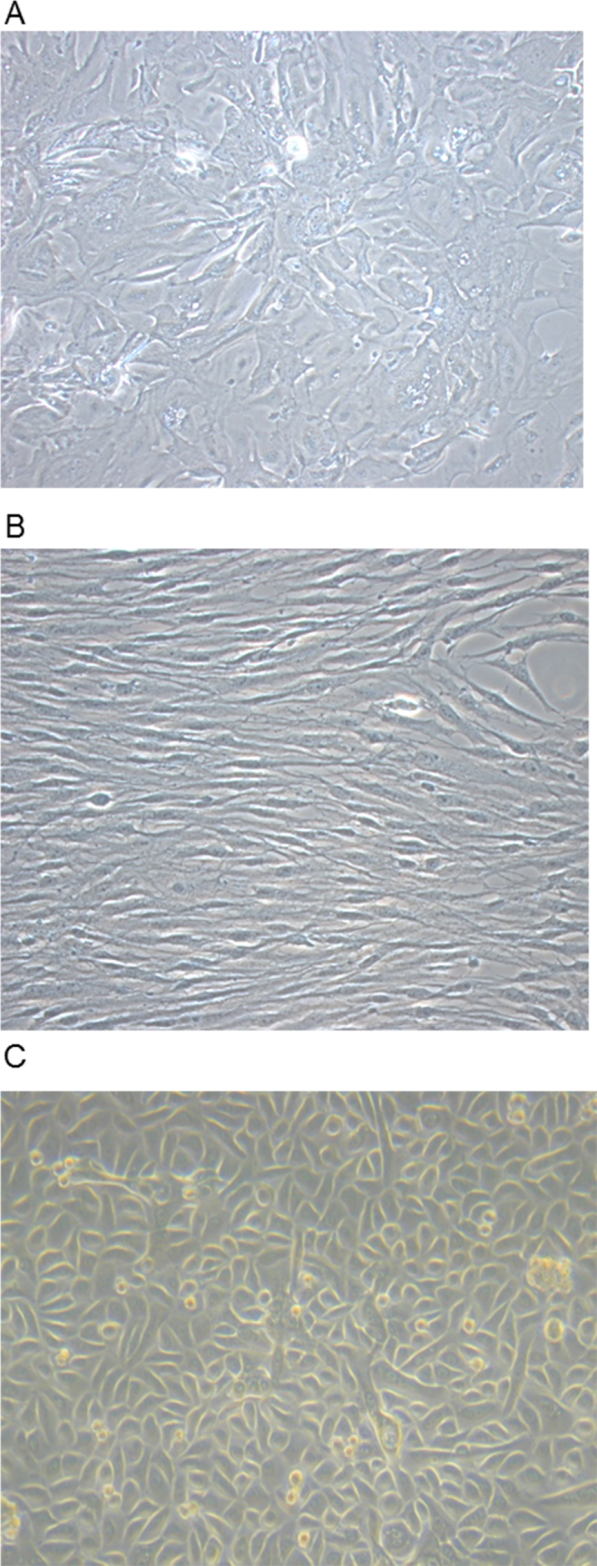
Morphology of mesenchymal stem cells. Phase contrast pictures of primary human mesenchymal stem cells (A), fibroblasts (B), and alveolar epithelial type II cells (C). Magnification x 20.

**Fig 2:**
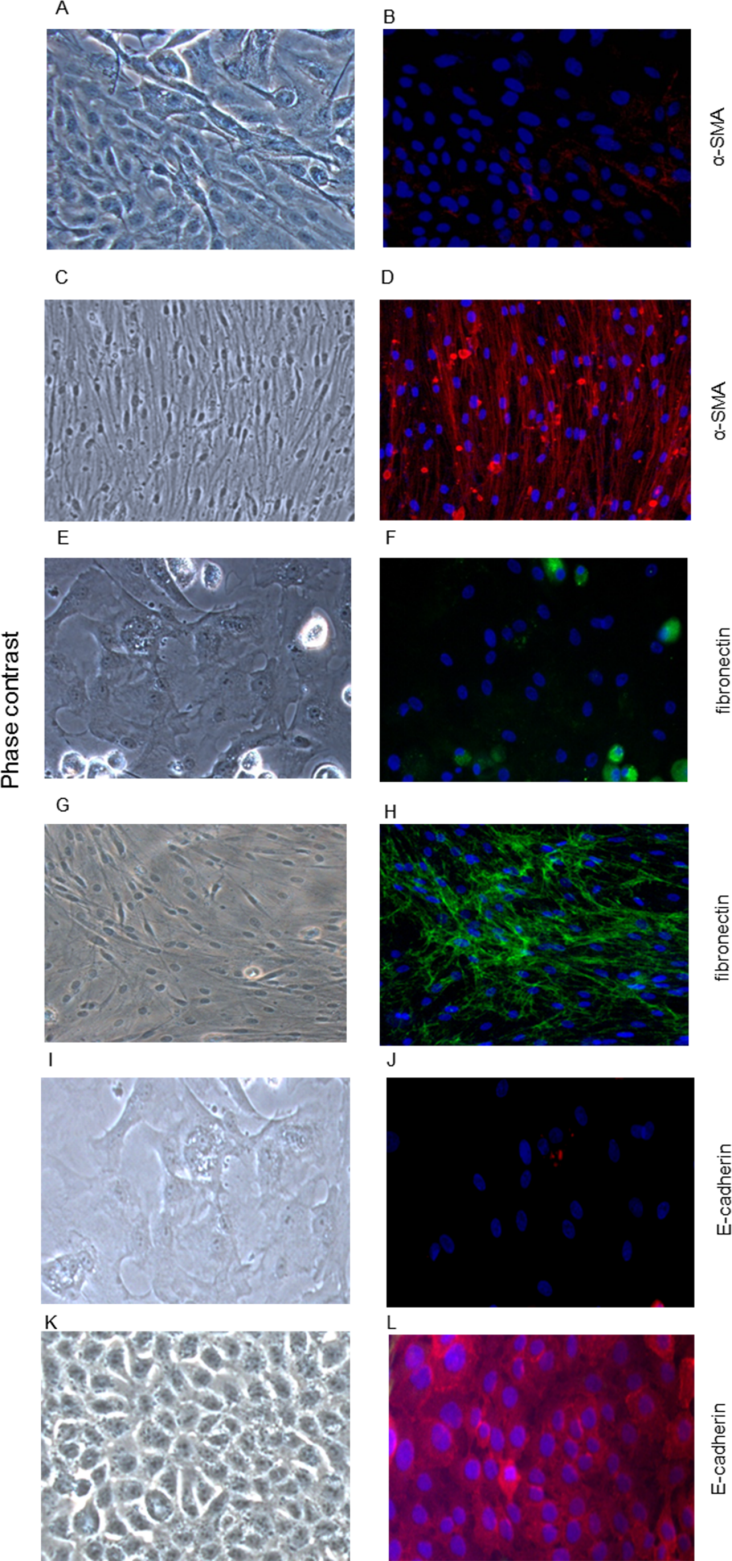
Characterization of mesenchymal stem cells. Primary human mesenchymal stem cells (A, B, E, F, I, J), fibroblasts (C, D, G, H), and alveolar epithelial cells (K, L) immunostained for α-smooth muscle actin (B, D), fibronectin (F, H), and E-cadherin (J, L). Corresponding phase contrast pictures are shown in panels A, C, E, G, I, and K. Cells were grown to confluence in normal growth medium, were then fixed, and permeabilized. Primary antibodies were detected by addition of fluorescein-labelled (green) or Cy3-labelled (red) secondary antibodies. Visualization by fluorescence microscopy. Magnification x 20.

**Fig 3:**
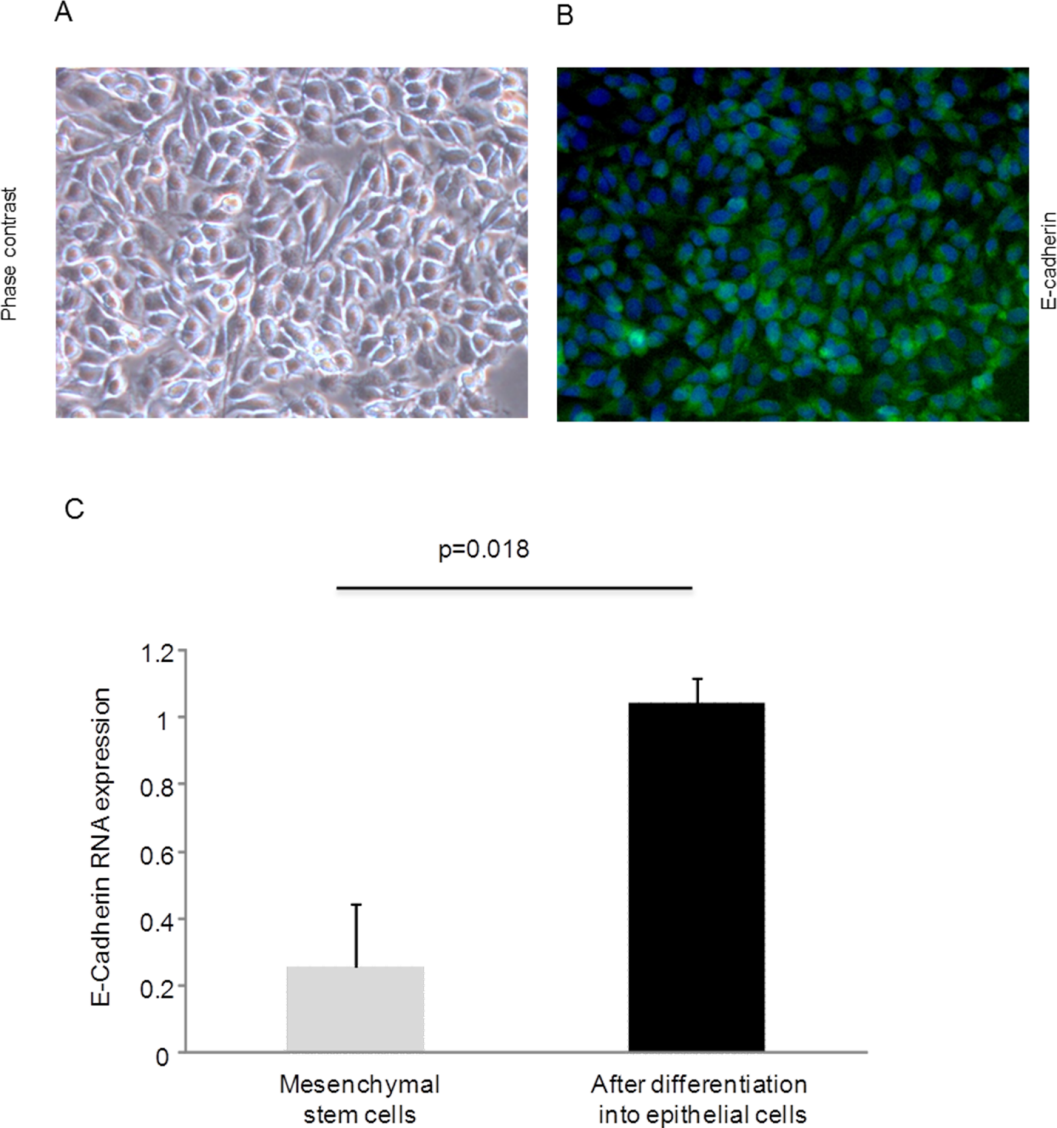
Epithelial differentiation of mesenchymal stem cells. Primary human mesenchymal stem cells were cultured in an epithelial growth medium (Cnt-17, CellnTEC Advanced Cell System AB) for 3–4 days, cells were fixed, and permeabilized. Cells were immunostained for E-cadherin (B). The primary antibody was detected by addition of a fluorescein-labelled (green) secondary antibody. (A) Corresponding phase contrast picture. Visualisation by fluorescence microscopy. Magnification x 20. (C) E-cadherin RNA expression in mesenchymal stem cells before (grey bar) and after (black bar) epithelial differentiation. RNA expression was assessed by quantitative real time RT-PCR. Data are presented as mean ± SEM of independent experiments performed in three different cell lines.

Cell cultures were established from lung tissue obtained from 32 patients. The characteristics of these patients are shown in Table 1. Indication for lung biopsy was lung volume resection surgery (LVRS) due to emphysema/bullectomy in 8/32 (25%) patients, lung resection due to benign or malign lung tumor in 6/32 (19%) patients, diagnostic biopsy due to chronic cough of unknown origin in one patient (3%), and in 17/32 (53%) patients lung biopsy was part of a diagnostic work-up due to non-classified fibrotic lung disease. Two to eight days after the start of culture (using supplemented RPMI) distinct cells started to sprout and grow out of the biopsy pieces and reached confluence around the biopsy after eight to ten days. As shown in Fig 1A, these cells exhibited neither the typical spindle-shape morphology of fibroblasts (Fig 1B), nor the typical cobble-stone morphology of alveolar epithelial type II cells (Fig 1C). In contrast, the cells showed an intermediate morphology with heterogeneity in shape. Therefore, we termed them *intermediate cells* until final characterization. In contrast to primary human lung fibroblasts (Fig 2D and 2H), immunofluorescence studies in these intermediate cells demonstrated only weak stainings for the mesenchymal markers α-SMA (Fig 2B) and fibronectin (Fig 2F). Likewise, compared to primary human alveolar epithelial type II cells (Fig 2L), intermediate cells showed only weak E-cadherin staining (Fig 2J). When culture medium of confluent intermediate cells was changed from RPMI containing 10% FCS to an epithelial growth medium (Cnt-17, CELLnTEC Advanced Cell System AB), cells started to change their shape into a cobble-stone morphology and after approximately 72 hours the cells were morphologically consistent with alveolar epithelial cells (Fig 3A). The differentiation into epithelial cells was demonstrated by positive immunofluorescence staining for E-cadherin (Fig 3B) and confirmed by real time RT-PCR showing significantly enhanced E-cadherin mRNA expression after epithelial differentiation as compared to a low E-cadherin mRNA expression in intermediate cells (Fig 3C).

### Undifferentiated cells are stem cells and originate from the bone marrow

**Fig 4:**
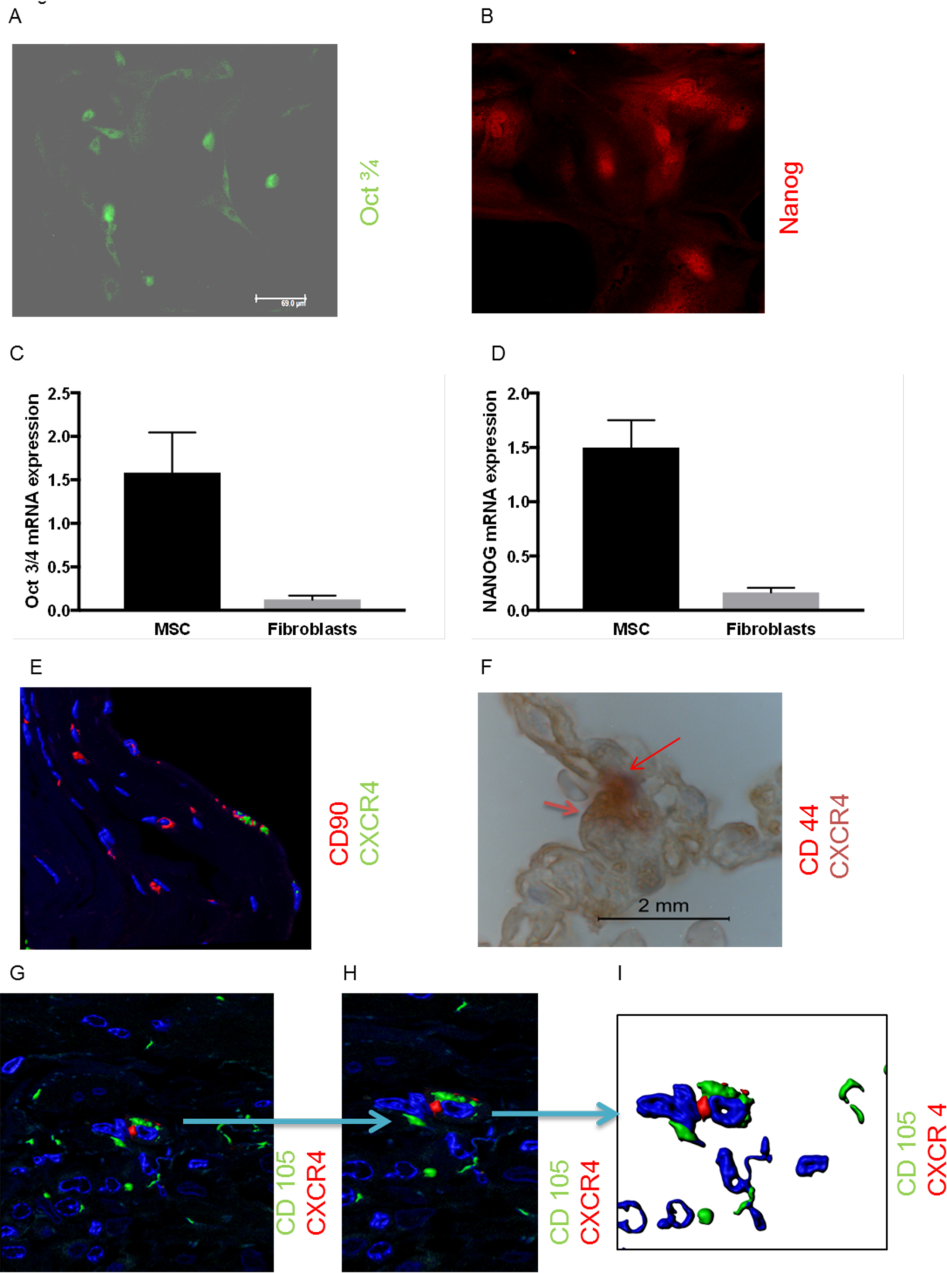
Characterization of mesenchymal stem cells. Primary human mesenchymal stem cells immunostained for Oct3/4 (A), and Nanog (B). Cells were fixed and permeabilized. Primary antibodies were detected by addition of fluorescein-labelled (FITC) (green) and Alexa Fluor 594-labelled (red) secondary antibodies. E, G-I: Double immunofluorescence staining for CD90 (E), CD105 (G, H, I) and C-X-C-chemokine receptor type 4 (CXCR4) (panels E, G, H, I) in lung tissue sections from patients with histologically confirmed IPF/UIP and chronic fibrotic hypersensitivity pneumonitis. Primary antibodies were detected by addition of secondary antibodies labelled with fluorescein FITC (green) for CD105 and CXCR4 detection or Cy3-labelled (red) for CD90 and CXCR4 detection. Magnification x40. Images were acquired using LSM 510 confocal microscope. Panel H and I is a 3D reconstruction to show more clear co stainings on the same cell. F: Co-staining with CD44 (pink) and CXCR4 (dark red) (Scale bar 2μm, Magnification x100 (oil)). (C, D) Oct3/4 (C) and Nanog (D) mRNA expression in mesenchymal stem cells (black bars) and in fibroblasts (grey bars). RNA expression was assessed by quantitative real time RT-PCR. Data are presented as mean ± SEM of independent experiments performed in three different cell lines.

To further characterize these cells the presence of the pluripotency markers, Oct3/4 and Nanog, was determined. Immunofluorescence staining for Oct3/4 and Nanog was positive in the cells (Fig 4A and 4B) indicating that these cells are possibly pluripotent. As a control, Oct3/4 staining was performed in primary human lung fibroblasts, but no positive signal could be observed (S1 Fig). Additional real time RT-PCR data confirmed the expression of Oct3/4 and Nanog mRNA in intermediate cells as compared to a low expression in fibroblasts (Fig 4C and 4D). To further investigate the allocation of these cells *in vivo*, lung tissue sections from patients with histologically confirmed IPF/UIP and chronic fibrotic hypersensitivity pneumonitis were used for further immunofluorescence/immunohistochemistry studies. Distinct cells with positive staining for the MSC markers CD90 (Fig 4E), CD44 (Fig 4F) and CD105 (Fig 4G–4I) were observed in close proximity to the alveolar epithelium, identifying the cells of interest as MSC. The bone marrow as the origin of the cells was suggested in the lung tissue sections by positive co-staining for C-X-C-chemokine receptor type 4 (CXCR4) (Fig 4E, 4F and 4G). Additional staining data have been published previously [13].

### Stem cells have the ability for mesenchymal differentiation *in vitro*

**Fig 5:**
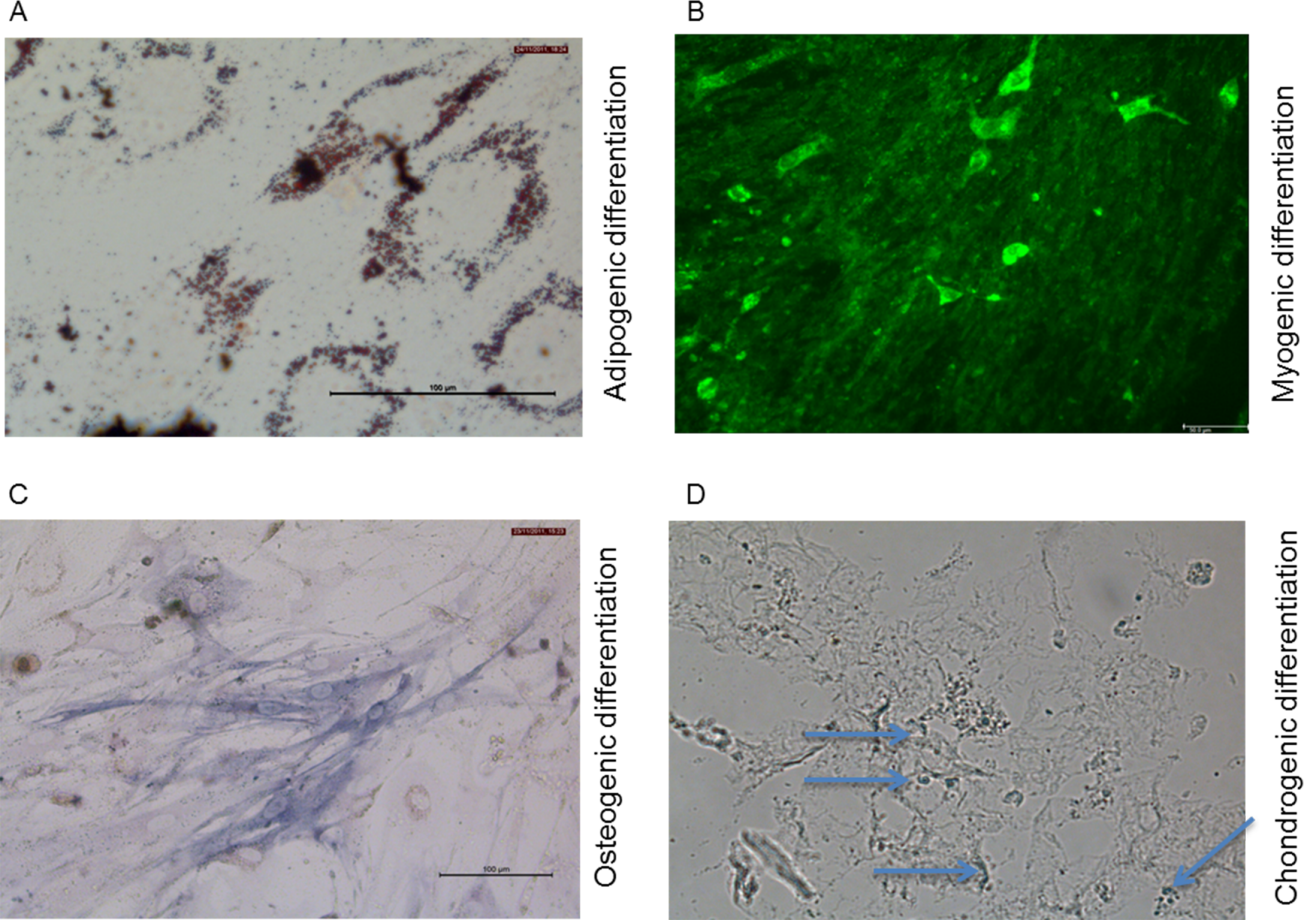
Mesenchymal differentiation of mesenchymal stem cells. Differentiation of stem cells into adipocytes (A), myofibroblasts (B), osteoblasts (C), and chondroblasts (D). Adipogenic differentiation was assessed with Red oil O staining for fat vacuoles (A); Myogenic differentiation was assessed via α-SMA-staining (B); Osteogenic differentiation was demonstrated by activity of alkaline phosphotase (C); Chondrogenic differentiation was demonstrated via Toluidine Blue-staining (D).

To further characterize the differentiation potential of the stem cells adipogenic, osteogenic, myogenic, and chondrogenic differentiation was induced in these cells. As demonstrated in Fig 5A, the cells were able to undergo adipogenic differentiation demonstrated by positive red oil O stains for fat vacuoles. The myogenic differentiation was shown by positive staining for α-SMA (Fig 5B), and the positive staining for alkaline phosphatase demonstrated the osteogenic differentiation (Fig 5C). The chondrogenic differentiation was proven by positive staining for toluidine blue (Fig 5D). Control experiments for osteogenic, chondrogenic, and adipogenic differentiation potential were performed using primary human lung fibroblasts, but no specific differentiation was observed (S2 Fig).

### Mesenchymal stem cells express hepatocyte growth factor

Biologically relevant levels of anti-fibrotic HGF (2124.9 ± 118 pg/ml) were measured in MSC-CM.

### Effect of mesenchymal stem cells conditioned media on cell proliferation and wound healing

**Fig 6:**
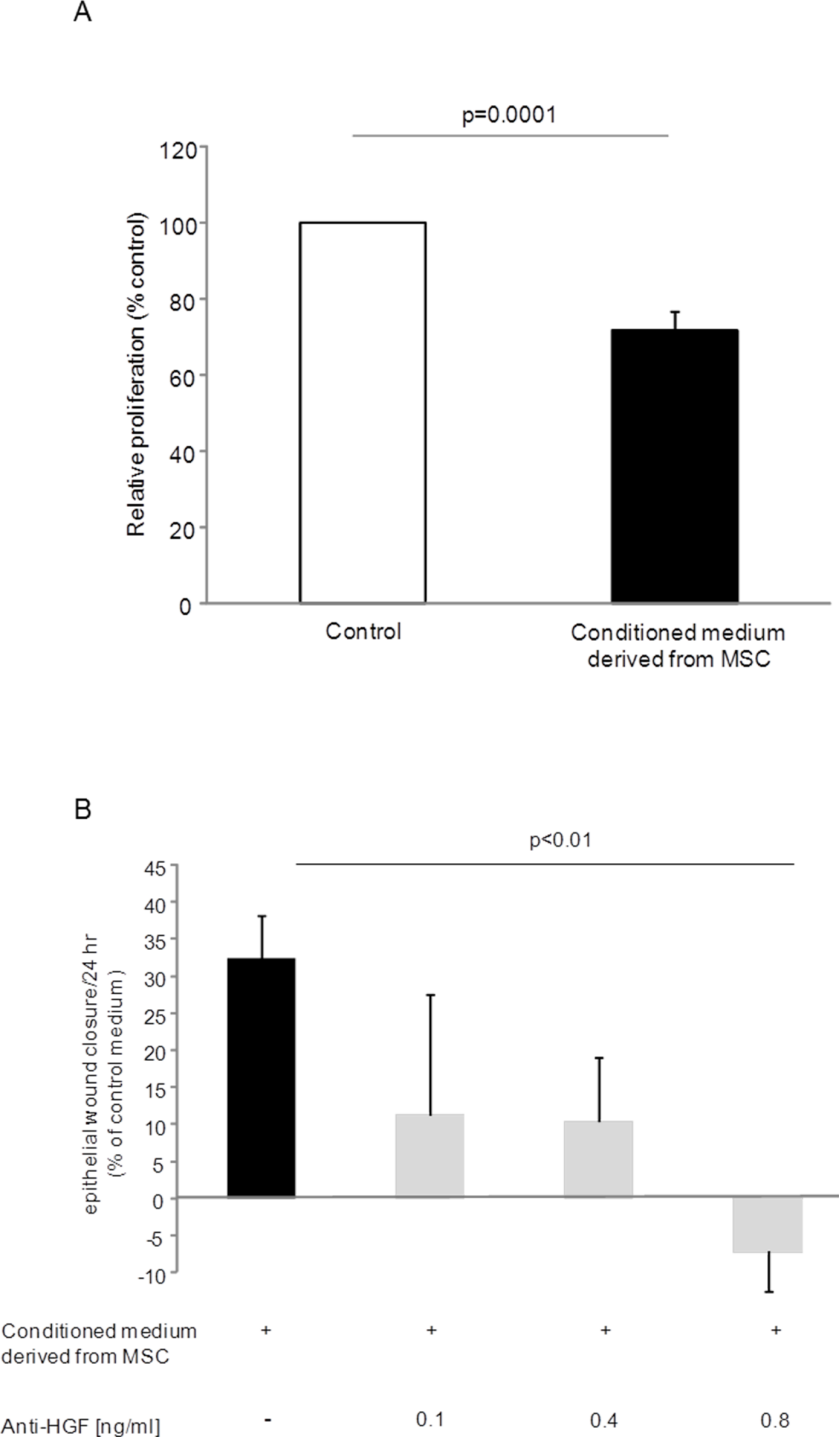
*In vitro* effects of mesenchymal stem cells. (A) Effect of conditioned medium derived from mesenchymal stem cells (MSC) (black bar) on fibroblast proliferation compared to control medium (open bar). Normal primary human lung fibroblasts (n = 2) were stimulated with MSC-derived conditioned medium from 8 different subjects. Data are presented as mean ± SEM of independent experiments. (B) Effect of MSC-derived conditioned medium (black bar) on epithelial wound closure after mechanical injury and dose-dependent effect of anti-hepatocyte growth factor (HGF) antibodies (grey bars). Bars represent means ± SEM expressed as percentage change from control medium.

To assess possible anti-fibrotic properties of MSC, the effect of MSC-CM on fibroblast proliferation and on lung epithelial wound repair was studied. As demonstrated in Fig 6A, MSC-CM (n = 8) significantly inhibited primary human lung fibroblast proliferation (29% growth inhibition, p = 0.0001). The epithelial wound repair capacity of wounded A549 epithelial cells incubated with MSC-CM was analyzed and compared to wounded A549 cells incubated with control medium. Compared to control medium (= 0% wound closure), alveolar epithelial wound closure was increased in the presence of MSC-CM at 24 hours (+ 32.4% wound closure, Fig 6B). This positive repair effect was abolished in the presence of 0.8 ng/ml neutralizing anti-HGF-antibodies (p = 0.006, Fig 6B). Control experiments for epithelial wound closure using conditioned medium derived from primary human lung fibroblasts were performed (S3 Fig).

### Increased numbers of mesenchymal stem cells in adult human lung tissue derived from patients with fibrotic lung diseases compared to non-fibrotic control lungs

**Fig 7:**
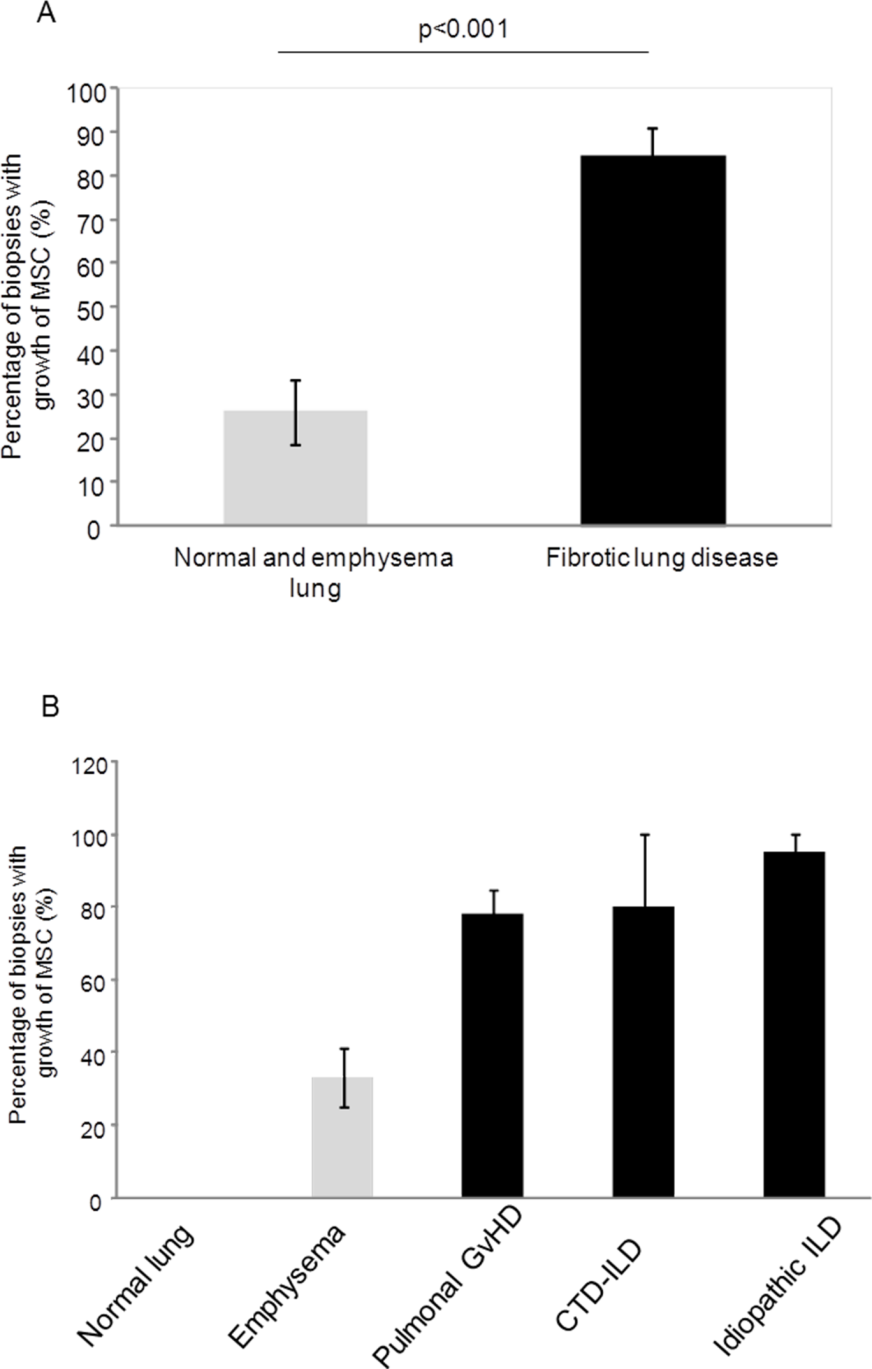
Outgrowth-rate of mesenchymal stem cells from biopsies derived from human lung tissue. (A) Percentage of biopsies with growth of mesenchymal stem cells (MSC) in lung tissue derived from fibrotic lungs (black bar) as compared to emphysema/normal lung (grey bar). (B) Percentage of biopsies with growth of MSC subdivided according to histological diagnosis. Bars represent means ± SEM expressed as [number of biopsies with growth of MSC / total number of biopsies set up]. GvHD: graft-versus-host disease; CTD: connective tissue disease; ILD: interstitial lung disease.

Cell culture for MSC was set up from 32 different lung specimens obtained from 32 patients undergoing lung biopsy for different reasons (Table 1). In 12 (37.5%) of those 32 patients the final histological diagnosis of the lung tissue used for cell culture was lung emphysema and in three patients (9%) normal lung tissue was used for cell culture (Table 2). Pulmonal graft-versus-host disease (GvHD) after hematopoietic stem cell transplantation (HSCT) with constrictive/obliterative or lymphocytic bronchiolitis was found in six (19%), usual interstitial pneumonia (UIP) in four (12.5%) and organizing pneumonia (OP) in two (6%) patients (Table 2). In 5 patients (16%) with connective tissue diseases (CTD) various fibrotic changes were found on histology. Table 2 summarizes the histological and chest computed tomography findings of all 32 patients. In total 95 biopsy-pieces were set up from the 17 patients with fibrotic lung diseases and 111 biopsy-pieces from the 15 patients with emphysema or normal lung. Analysis of positive MSC out-growth revealed that in 85% of set up biopsies derived from fibrotic lung tissue there was spontaneous out-growth of MSCs which was significantly more (p<0.001) as compared to 26% from emphysema and normal lung tissue (Fig 7A). When further dissecting the results according to histological diagnosis it showed that there was no MSC growth in normal lung, whereas in the emphysema growth of MSC was observed in 33% of set up biopsies (Fig 7B). In lungs derived from patients with pulmonal GvHD after HSCT MSC outgrowth was positive in 78% of set-up biopsies, in lung tissue obtained from patients with CTD-associated interstitial lung diseases in 80%, and in lung tissue derived from patients with idiopathic interstitial lung diseases (OP, UIP/IPF) a MSC growth-rate of 95% was observed (Fig 7B). Importantly, the lack of MSC growth from normal control lung was confirmed by absence of immunofluorescence stainings for CD44, CD90, and CD105 in paraffin embedded lung slices derived from normal lung tissue (S4 Fig).

**Table 2:** Computed tomography findings, type of biopsy, histologic findings of lung tissue used for cell culture, final diagnosis, and percentage of positive mesenchymal stem cell outgrowth.

Patient number	Age	Sex	High resolution computed tomography findings	Surgical lung biopsy (SLB) versus transbronchial biopsy (TBB)	Histological diagnosis of tissue used for cell culture	Final multidisciplinary diagnosis	Number of biopsies with growth of MSC / total number of biopsies set up [%]		
2	47	f	Pulmonary nodule right upper lobe, 9 x 7 mm	SLB	Normal alveolar lung tissue	Hamartoma	0	Normal lung	
16	70	m	Multiple pulmonal nodules	SLB	Normal alveolar lung tissue	Lung metastasis of urothel carcinoma	0		
27	61	f	No abnormal findings	TBB	Normal alveolar lung tissue	Chronic cough of unknown origin	0		
1	80	m	Lung emphysema with bulla in middle lobe	SLB	Emphysema	COPD with emphysema	83	Emphysema	
10	56	m	Centriacinar emphysema in both lungs	SLB	Emphysema	COPD with emphysema	0		
11	51	m	Panacinar and paraseptal emphysema in both lungs	SLB	Emphysema	COPD with emphysema	50		
13	66	f	Bullous emphysema in both lungs	SLB	Emphysema	COPD with emphysema	0		
14	68	f	Emphysema with basal predominance in both lungs	SLB	Emphysema	COPD with emphysema	33		
17	73	m	Bullous emphysema in both lungs	SLB	Emphysema	COPD with emphysema	56		
18	55	f	Centriacinar emphysema in both lungs	SLB	Emphysema	COPD with emphysema	43		
19	79	m	Multiple nodules in the right lung	SLB	Emphysema	Emphysema/NSCLC	0		
20	66	m	Bullous emphysema in both lungs	SLB	Emphysema	COPD with emphysma	0		
21	65	m	Panlobular emphysema in both lungs	SLB	Emphysema	COPD with emphysema	57		
24	74	f	Centriacinar emphysema and multiple nodules in both lungs	SLB	Emphysema	COPD with emphysema/NSCLC	19		
25	72	f	Centriacinar and panlobular emphysema in both lungs; Consolidation in right upper lobe	SLB	Emphysema	COPD with emphysema/NSCLC	50		
5	57	m	Ground glass abnormalities in the right lung	SLB	Obliterative bronchiolitis and organizing pneumonia	Pulmonal GvHD with BO after HSCT	58	Pulmonal GvHD after HSCT	Fibrotic lung diseases
6	29	m	Multiple nodules in both lungs	SLB	Constrictive obliterative bronchiolitis	Pulmonal GvHD with BO after HSCT	100		
7	41	m	Normal lung parenchyma	SLB	Constrictive obliterative bronchiolitis	Pulmonal GvHD with BO after HSCT	60		
12	55	m	Peribronchiolar ground glass opacities and consolidations in both lungs; Tree-in-bud pattern in both lungs	SLB	Lymphozytic bronchiolitis with supepithelial fibrosis, organizing pneumonia	Pulmonal GvHD with lymphocytic bronchiolitis after HSCT	80		
15	65	m	Ground glass opacities and consolidations in the left lung, hypoperfused areas in both lungs	SLB	Lymphozytic bronchiolitis with mucus retention, subepithelial fibrosis	Pulmonal GvHD with lymphocytic bronchiolitis after HSCT	80		
32	60	m	Peribronchiolar ground glass opacities, centrilobular nodules and subpleural consolidations	SLB	Lymphozytic bronchiolitis, endothelialitis of pulmonal arterioles, and subpleural fibrosis	Pulmonal GvHD with lymphocytic bronchiolitis after HSCT	90		
4	34	f	Subpleural fibrotic changes with basal predominance in both lungs	TBB	Lung parenchyma with retention of alveolar macrophages	Systemic sclerosis with lung involvement	100	CTD-associated ILD	
8	81	m	Subpleural honeycombing and ground glass abnormalities in both lungs	SLB	Peribronchovascular, alveolo-septal fibrosis	Rheumathoid arthritis with lung involvement	0		
9	47	f	Consolidations, ground glass abnormalities, and bronchiectasis in right lung	TBB	Alveoloseptal lymphocytic inflammation	Rheumathoid arthritis with lung involvement/NSIP	100		
22	59	f	Normal lung parenchyma	TBB	Respiratory bronchiolitis-interstitial lung disease	Lupus erythematodes with lung involvement	100		
26	74	f	Reticular changes and ground glass abnormalities, compatible with NSIP	TBB	Alveolo-septal fibrosis with lymphozytic inflammation	Lupus erythematodes with lung involvement/NSIP	100		
3	65	f	Consolidations, ground glass abnormalities, and bronchiectasis in both lungs	TBB	Organizing pneumonia	Cryptogenic organizing pneumonia	100	Idiopathic ILD	
23	84	f	Reticular abnormalities, bronchiectasis	TBB	Organizing pneumonia	Cryptogenic organizing pneumonia	100		
28	72	m	Subpleural reticular abnormalities, tranction bronchiectasis	SLB	Usual interstitial pneumonia	Idiopathic pulmonary fibrosis	100		
29	60	f	Usual interstital pneumonia pattern: reticular abnormalities, honeycombing, traction bronchiectasis, basal predominance	SLB	Usual interstitial pneumonia	Idiopathic pulmonary fibrosis	100		
30	59	m	Reticular abnormalities and honeycombing in both lungs	SLB	Usual interstitial pneumonia	Idiopathic pulmonary fibrosis	100		
31	56	m	Reticular abnormalities with subpleural and basal predominance	SLB	Usual interstitial pneumonia	Idiopathic pulmonary fibrosis	70		

Grouping of patients according to histologic findings of lung tissue used for cell culture. COPD: chronic obstructive pulmonary disease; CTD: connective tissue disease; GvHD: graft-versus-host disease; HSCT: hematopoietic stem cell transplantation; ILD: interstitial lung disease; NSCLC: non-small cell lung cancer; SLB: surgical lung biopsy; TBB: transbronchial biopsy.

In this study we provide evidence that fibrotic adult human lung contains stem/progenitor cells. The identified cells express markers of mesenchymal origin and are able to differentiate towards a mesenchymal phenotype, thus we defined them as mesenchymal stem cells (MSC). The secretome obtained (MSC-CM) inhibits the proliferation of fibroblasts and enhances HGF-mediated lung epithelium wound repair *in vitro*. Furthermore, significantly enhanced numbers of MSC were grown from lung tissue obtained from patients with fibrotic lung diseases as compared to lung tissue obtained from patients with emphysema or with normal lung.

Presence of stem/progenitor cells in the lung and their role in health and disease has been discussed [19, 20]; the nomenclature or classification of these cells has been a matter of debate and is still not fully agreed [21–23].

As generally accepted, MSC are self-renewing cells characterized by i) plastic adherence, ii) expression of CD44, CD73, CD90 and CD105, iii) lack of expression of hematopoietic markers, and iv) their ability to differentiate into multiple cell types such as adipocytes, osteocytes and chondrocytes [24]. In accordance with previous reports for the characterization of MSC [25, 26], the cells of interest isolated from the lung stained positive for the mesenchymal markers CD44, CD90, CD105. Additionally, the cells had the capacity to differentiate into adipocytes, osteocytes and chondrocytes, identifying them as MSC.

Intriguingly, we found that lung-derived MSC also express markers of pluripotency OCT3/4 and Nanog and they also have the ability to differentiate into epithelial cells which is in agreement with previous studies [6, 27, 28]. Based on our findings we speculate that MSC play a pivotal role in injury and repair mechanisms of the lung by replacing damaged epithelium. Our hypothesis is supported by earlier data showing engraftment of MSC at sites of lung injury and differentiation into epithelial cells [6, 29].

Complementary to the *in vitro* data from isolated cells we were able to detect MSC in lung tissue sections derived from patients with IPF and chronic hypersensitivity pneumonitis, localized in the interstitium close to the alveolar epithelium. Co-staining for CXCR4 with CD44, CD90 or CD105 suggested the origin of these cells from the bone marrow and supports previous studies showing that bone marrow-derived MSC actively migrated to damaged tissue [30, 31].

The exact role of MSC in the lung is still debated, yet evidence suggests towards their pivotal role in repair mechanisms [32]. Several studies have shown a significant benefit of MSC administration in different organs such as bone, heart, and ischemic brain [33–35]. Similarly, for lung diseases various animal models demonstrated beneficial effects of exogenously administrated stem cells after lung injury, such as reduction of collagen deposition and improvement of lung repair [5, 7–9, 13, 36]. In contrast, an increased expression of α-SMA and collagen I by MSC isolated from BAL of patients with bronchiolitis obliterans syndrome has been demonstrated, possibly representing a pro-fibrotic phenotype which contributes to fibrogenesis [37]. However, these *in vitro* data lack further proof of functional pro-fibrotic effects and are in contrast to a considerable number of *in vivo* data demonstrating a beneficial effect of stem cells with regard to tissue fibrosis. Furthermore, direct comparisons between the aforementioned study and our own should be done with caution, as Walker et al isolated their MSC from BAL fluid, whereas we cultured our cells from biopsies. In our study MSC-CM increased lung epithelial wound repair, which was mediated by hepatocyte growth factor (HGF). Moreover, MSC-CM significantly inhibited the proliferation of primary human lung fibroblasts. Thus, we demonstrate a significant anti-fibrotic effect of the MSC-secretome *in vitro*. This is in line with our previous report where HGF-expressing BMSC cells attenuated bleomycin-induced pulmonary fibrosis in a rat model by increasing alveolar epithelial cell proliferation and reduction of myofibroblasts [13].

Analysis of MSC-outgrowth from lung tissue revealed that significantly more multipotent MSC grew from biopsies derived from patients with fibrotic lung diseases, mainly IPF, OP, and BO, as compared to emphysema or normal lung tissue. Interestingly, there was no MSC-growth observed in the normal lung tissue, whereas in emphysema lungs MSC-outgrowth was detected in 33% of all biopsies set up. Our *in vitro* findings were further supported by the absence of MSC in lung tissue slices obtained from normal lung tissue, even though further studies with more extensive tissue sampling will be needed to consolidate our findings in normal lung tissue. Our data is in agreement with Horwitz E et al. who stated that there is little evidence for the homing of MSC to healthy tissue [38]. In contrast to our study Sinclair et al. recently showed that MSC with a surface marker expression profile similar to the one of the cells we isolated in this study can be readily isolated from healthy lung tissue and from BAL of lung transplant recipients [39]. Interestingly, MSC isolated from BAL, despite of expressing similar surface markers, showed different gene expression and differentiation potential as compared to MSC isolated from lung tissue [39]. This supports the notion that several unique MSC populations with a similar phenotype exist within the lung. MSCs described in our study are suggested to originate from the bone marrow and are located in the interstitial area and may therefore represent a not yet described population of MSC within the lung. Our observation of increased numbers of MSC in adult human lung from patients with fibrotic lung diseases as compared to non-fibrotic lung tissue supports prior animal studies by Rojas et al. using the bleomycin-model [5]. Based on our findings, we hypothesize that bone marrow-derived MSC are attracted to areas of tissue injury where they exert anti-fibrotic effects by inhibition of fibroblast proliferation and inducing alveolar wound repair capacity in a paracrine manner via HGF. To prove this hypothesis, additional *in vivo* animal models will be indispensable. Inevitable the question arises why—in the presence of enhanced numbers of potentially anti-fibrotic MSC—patients still develop fibrotic lung diseases. The recent study by Tashiro et al. might provide an explanation, as they have demonstrated that old MSC lose their anti-fibrotic properties [7]. However, whether the here-described anti-fibrotic effects of MSC are age-dependent or due to altered microenvironment in *in-vitro* culture remains to be studied.

We acknowledge that the present study has some limitations. The lung tissue used for culture of MSC derived from a mixed population of patients with fibrotic lung diseases. It might have been more concise to study e.g. only lung tissue from patients with IPF, however, due to the new evidence-based guidelines for diagnosis of IPF where patients with a classical UIP pattern on CT scan do not need surgical lung biopsy, access to IPF lung tissue becomes challenging [40]. In contrast, the source of our data strengthens our hypothesis that MSC might support a disease-independent, general injury-repair process.

## Conclusions

In summary, our study demonstrates enhanced numbers of multipotent MSC in fibrotic lung tissue as compared to emphysema and normal lung. The cells’ anti-fibrotic properties *in vitro* makes them an interesting candidate to be tested as a novel therapeutic approach for patients with IPF and further *in vivo* studies are needed to consolidate such an approach.

## Supporting information

S1 Fig Oct3/4 staining in fibroblasts. Negative immunofluorescence staining for Oct3/4 and corresponding phase contrast picture in primary human lung fibroblasts. Cells were fixed and permeabilized. Primary antibody was detected by addition of fluorescein-labelled (FITC) (green) secondary antibody. Magnification x 20.
(DOCX)

S2 Fig Mesenchymal differentiation assay in fibroblasts. Negative differentiation assays of primary human lung fibroblasts into adipocytes (A), chondroblasts (B), and osteoblasts (C). Adipogenic differentiation was assessed with Red oil O staining for fat vacuoles (A); Chondrogenic differentiation was demonstrated via Toluidine Blue-staining (B). Osteogenic differentiation was demonstrated by activity of alkaline phosphotase (C).
(DOCX)

S3 Fig Effect of conditioned medium derived from fibroblasts on epithelial wound closure after mechanical injury. Epithelial wound repair capacity of wounded A549 epithelial cells incubated with fibroblast-derived conditioned medium was assessed and compared to wounded A549 cells incubated with control medium. Effect of control medium = 0% wound closure. Bar represent means ± SEM expressed as percentage change from control medium.
(DOCX)

S4 Fig CD44, CD90, and CD105 staining in normal human lung tissue. Negative immunofluorescence stainings for CD44 (A), CD90 (B), and CD105 (C) in lung tissue sections from patients with histologically normal lung tissue. Formalin-fixed and paraffin-embedded lung tissue was used. Primary antibodies were detected by addition of secondary antibodies labelled with FITC (green) CD 44 and CD 105 or Cy3-labelled (red) for CD 90. The images were acquired using the LSM 510 confocal microscope. Magnification x20.
(DOCX)
