# Correction: Multipotent mesenchymal stem cells in lung fibrosis

**DOI:** 10.1371/journal.pone.0191144

**Published:** 2018-01-09

**Authors:** The PLOS ONE Staff

The following information is missing from the Funding section: This project was supported by a Sinergia-grant from the Swiss National Research Foundation (CRSII3 160704). The publisher apologizes for the error.
