# Supplementary figures and images for: Multipotent mesenchymal stem cells in lung fibrosis
Source: PLoS One. 2017 Aug 21;12(8):e0181946. doi: 10.1371/journal.pone.0181946 (PMC5565112; doi:10.1371/journal.pone.0181946)

# FIGURE S1


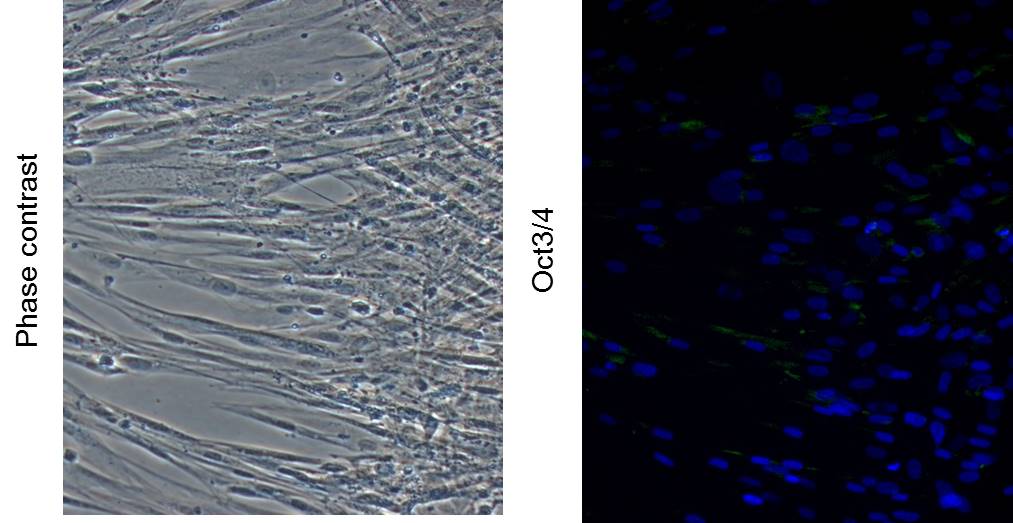

Supplement: S1 Fig — Negative immunofluorescence staining for Oct3/4 and corresponding phase contrast picture in primary human lung fibroblasts. Cells were fixed and permeabilized. Primary antibody was detected by addition of fluorescein-labelled (FITC) (green) secondary antibody. Magnification x 20. (DOCX) [file pone.0181946.s001.docx]

# FIGURE S2


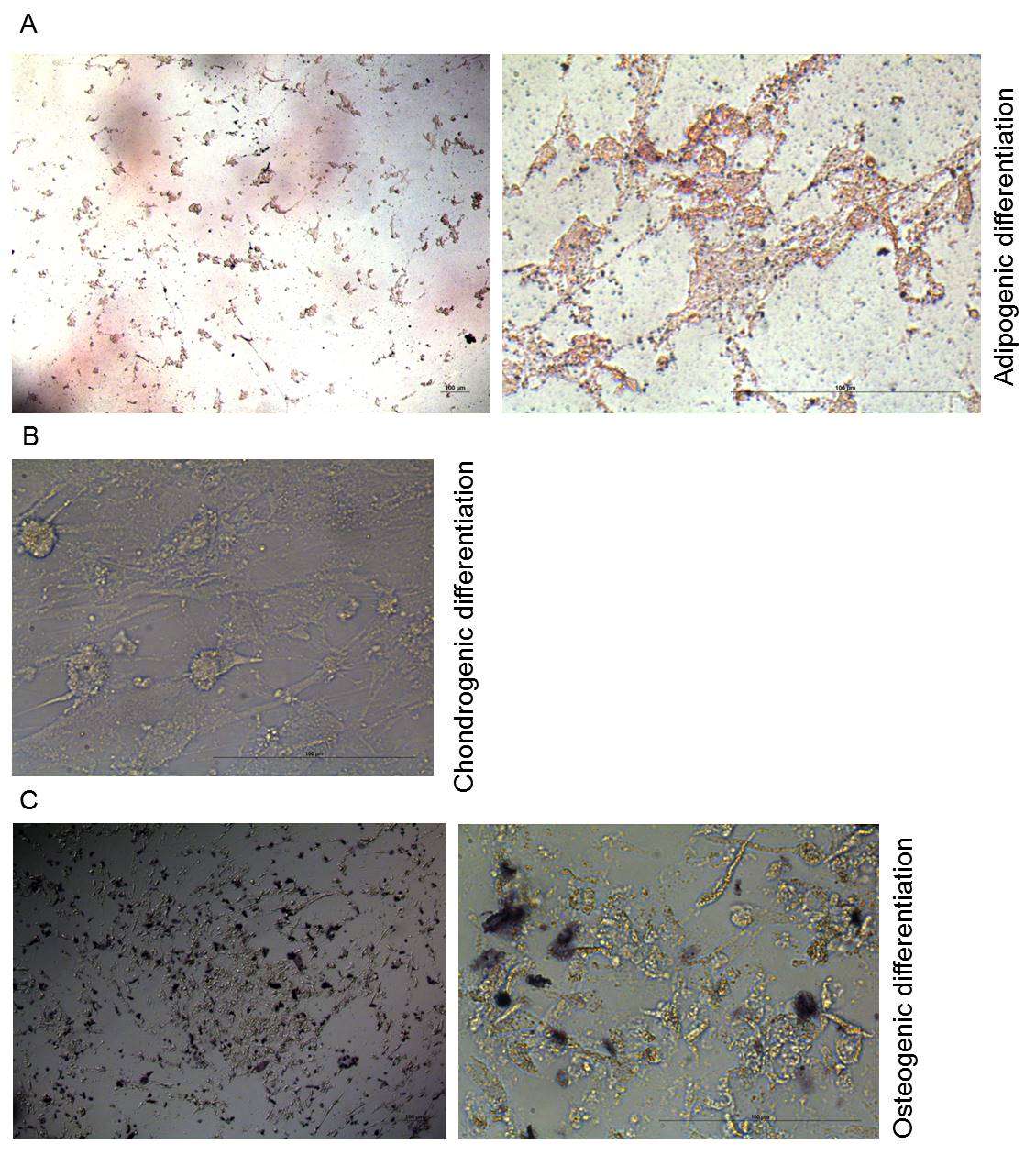

Supplement: S2 Fig — Negative differentiation assays of primary human lung fibroblasts into adipocytes (A), chondroblasts (B), and osteoblasts (C). Adipogenic differentiation was assessed with Red oil O staining for fat vacuoles (A); Chondrogenic differentiation was demonstrated via Toluidine Blue-staining (B). Osteogenic differentiation was demonstrated by activity of alkaline phosphotase (C). (DOCX) [file pone.0181946.s002.docx]

# FIGURE S3

*
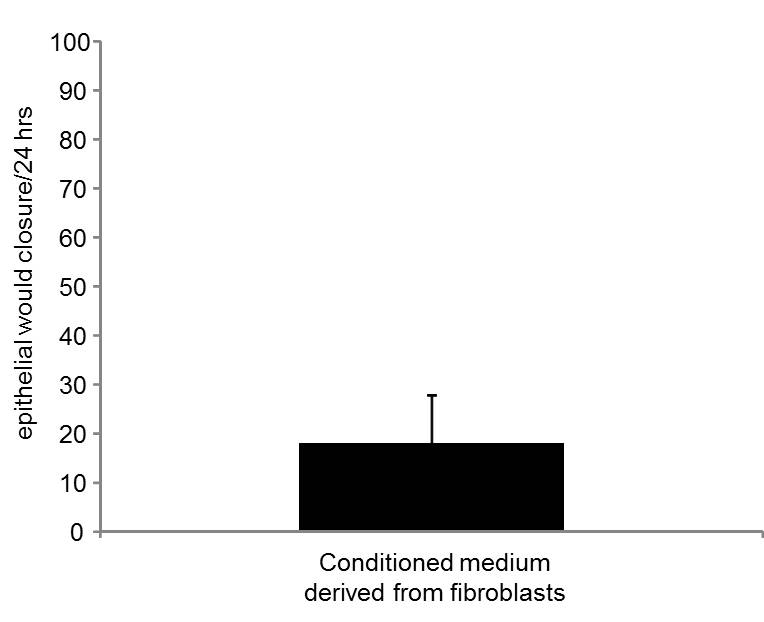
*

Supplement: S3 Fig — Epithelial wound repair capacity of wounded A549 epithelial cells incubated with fibroblast-derived conditioned medium was assessed and compared to wounded A549 cells incubated with control medium. Effect of control medium = 0% wound closure. Bar represent means ± SEM expressed as percentage change from control medium. (DOCX) [file pone.0181946.s003.docx]
