# Supplementary material for: Multipotent mesenchymal stem cells in lung fibrosis
Source: PLoS One. 2017 Aug 21;12(8):e0181946. doi: 10.1371/journal.pone.0181946 (PMC5565112; doi:10.1371/journal.pone.0181946)
Supplement: S4 Fig — Negative immunofluorescence stainings for CD44 (A), CD90 (B), and CD105 (C) in lung tissue sections from patients with histologically normal lung tissue. Formalin-fixed and paraffin-embedded lung tissue was used. Primary antibodies were detected by addition of secondary antibodies labelled with FITC (green) CD 44 and CD 105 or Cy3-labelled (red) for CD 90. The images were acquired using the LSM 510 confocal microscope. Magnification x20. (DOCX) [file pone.0181946.s004.docx]

FIGURE S4
